# A multisite exploration of the association between critical care implementation factors and clinical outcomes during the COVID-19 pandemic

**DOI:** 10.1017/cts.2023.22

**Published:** 2023-02-17

**Authors:** Santana Silver, Sarah Redmond, Kayla Christine Jones, Emily George, Sarah Zornes, Amelia Barwise, Aaron Leppin, Yue Dong, Lori A. Harmon, Vishakha K. Kumar, Christina Kordik, Mari-Lynn Drainoni, Allan J. Walkey

**Affiliations:** 1 Department of Medicine, Boston University School of Medicine, Evans Center for Implementation & Improvement Sciences (CIIS), 72 East Concord St, Boston, MA, 02118, USA; 2 Robert D. and Patricia E. Kern Center for the Science of Health Care Delivery, Mayo Clinic Rochester, 200 First Street SW, Rochester, MN 55905, USA; 3 Boston University School of Public Health, 715 Albany St, Boston, MA 02118, USA; 4 National Institutes of Health Post-baccalaureate Research Education Training, Patricia E. Kern Center for the Science of Health Care Delivery, Mayo Clinic Rochester, 200 First Street SW, Rochester, MN 55905, USA; 5 Division of Pulmonary and Critical Care Medicine, Mayo Clinic, 200 First Street SW, Rochester, MN, 55905, USA; 6 Mayo Center for Clinical and Translational Science (CCaTS), USA; 7 Department of Anesthesiology and Perioperative Medicine, Mayo Clinic, 200 First Street SW, Rochester, MN 55905, USA; 8 Department of Research and Quality, Society of Critical Care Medicine, 500 Midway Drive, Mount Prospect, IL 60056, USA; 9 Section of Infectious Diseases, Boston University School of Medicine, USA; 10 Department of Health Law, Policy & Management, Boston University School of Public Health, USA; 11 Department of Medicine, Evans Center for Implementation & Improvement Sciences (CIIS), Boston University School of Medicine, 801 Massachusetts Avenue, Room 2014, Boston, MA, 02118, USA; 12 The Pulmonary Center, Division of Pulmonary, Allergy, Critical Care and Sleep Medicine; Evans Center of Implementation and Improvement Sciences (CIIS), Department of Medicine, Boston University School of Medicine, 72 E. Concord St Housman (R), Boston, MA, 02118, USA

**Keywords:** COVID-19, critical care, implementation science, CFIR, barriers, facilitators

## Abstract

**Background:**

Little is known about strategies to implement new critical care practices in response to COVID-19. Moreover, the association between differing implementation climates and COVID-19 clinical outcomes has not been examined. The purpose of this study was to evaluate the relationship between implementation determinants and COVID-19 mortality rates.

**Methods:**

We used mixed methods guided by the Consolidated Framework for Implementation Research (CFIR). Semi-structured qualitative interviews were conducted with critical care leaders and analyzed to rate the influence of CFIR constructs on the implementation of new care practices. Qualitative and quantitative comparisons of CFIR construct ratings were performed between hospital groups with low- versus high-mortality rates.

**Results:**

We found associations between various implementation factors and clinical outcomes of critically ill COVID-19 patients. Three CFIR constructs (implementation climate, leadership engagement, and engaging staff) had both qualitative and statistically significant quantitative correlations with mortality outcomes. An implementation climate governed by a trial-and-error approach was correlated with high COVID-19 mortality, while leadership engagement and engaging staff were correlated with low mortality. Another three constructs (needs of patient; organizational incentives and rewards; and engaging implementation leaders) were qualitatively different across mortality outcome groups, but these differences were not statistically significant.

**Conclusions:**

Improving clinical outcomes during future public health emergencies will require reducing identified barriers associated with high mortality and harnessing salient facilitators associated with low mortality. Our findings suggest that collaborative and engaged leadership styles that promote the integration of new yet evidence-based critical care practices best support COVID-19 patients and contribute to lower mortality.

## Introduction

The coronavirus disease 2019 (COVID-19) pandemic created unprecedented challenges for health systems globally. Intensive care units (ICUs) were especially stressed with maintaining high-quality care for immense volumes of critically ill patients [1]. In response to COVID-19, critical care settings had to rapidly identify, access, and implement new practices. Yet, little is known about the strategies used by clinical leaders to select and integrate changing practices, the implementation determinants (i.e., organizational factors that facilitated and hindered implementation) of these practices, and how change management strategies and organizational characteristics influence clinical outcomes.

Prior qualitative research found that across critical care sites, collaborative leadership and communication mechanisms supported implementation of new practices during COVID-19 and buffered against implementation barriers like resource and staffing shortages [2]. However, *variation* in critical care contextual elements and, more importantly, the potential association between differing implementation climates and clinical outcomes has not been examined. Therefore, the purpose of this mixed methods study was to evaluate associations between differing implementation determinants across hospitals and clinical outcomes of critically ill patients during the COVID-19 pandemic.

## Methods

**Fig. 1.**
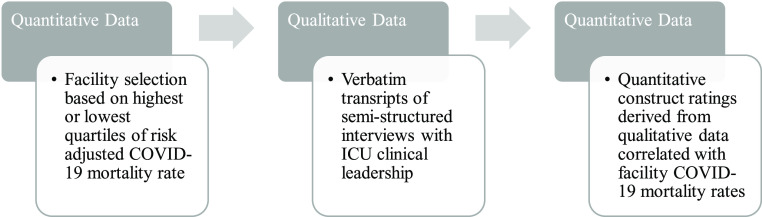
Mixed methods study design. ICU, intensive care unit.

We used mixed methods to investigate variability in implementation determinants through qualitative and quantitative data in order to determine what, if any, contrasts existed between US hospitals with large differences in mortality for critically ill patients with COVID [3]. Fig. 1 describes the quantitative and qualitative sequence, and how qualitative data were used and applied to assess clinical outcomes. Participant selection and qualitative data collection methods for this study have been previously described [2]. Briefly, twenty US hospitals, identified using an international COVID registry, were invited to participate in this study; ten each from higher and lower mechanically ventilated COVID-19 adjusted mortality quartiles [4,5,6]. ICU physician and nursing clinical leaders from each participating hospital were recruited and consented to participate in qualitative interviews using Zoom [7] video-conferencing software. Semi-structured interviews were conducted by EG to elicit open dialogue and probe for any critical care practice changes and implementation factors that may have impacted uptake of changes at participating hospitals [2]. The Boston Medical Center/Boston University Medical Center Institutional Review Board approved all study procedures. We followed the Consolidated Criteria for Reporting Qualitative Research checklist to develop the manuscript [8].

Quantitative and qualitative data were analyzed separately and then integrated to identify constructs within the Consolidated Framework for Implementation Research (CFIR) that were most strongly associated with hospital mortality rates due to COVID-19 [9]. We conducted this mixed methods approach in four major analytic stages: (1) *Qualitative analysis* of interview transcripts, driven by the CFIR; (2) *Quantification of qualitative data* using CFIR construct ratings; (3) *Qualitative comparison* of CFIR construct ratings between hospital outcome performance groups (i.e., hospitals from low versus high COVID-19 adjusted mortality quartiles); (4) *Quantitative comparison* of CFIR construct ratings between low- and high-mortality hospitals. ICU leaders were blinded as to whether they were at a low- or high-mortality hospital; analysts were blinded to risk-adjusted mortality quartile during qualitative analysis and quantification of qualitative data, after which analysts were unblinded to enable qualitative and quantitative comparisons between low- and high-mortality hospitals. Each stage is described below.

**(1) Qualitative analysis:** The guiding framework and consensual qualitative analytic approach used to guide the directed content analysis of qualitative interviews have been previously described [2,10,11]. Briefly, we used rapid evaluation methods [12,13,14,15] to identify and organize qualitative data and emerging themes into 14 salient CFIR constructs that have been demonstrated to influence implementation efforts [9]. Rapid template analysis [16] of interview transcripts consisted of three analysts (SS, KCJ, SR) coding the qualitative data using a CFIR-based coding framework and summarizing individual transcripts in templated summary tables.

**(2) Quantification of qualitative data:** After coding and transcript summaries were complete, the CFIR constructs in each templated summary table were subjected to a rating process. The evaluation team deliberated and reached consensus to assign a rating to each CFIR construct within each of the four transcripts that were initially double-coded. The ratings reflected the perceived influence each construct had on implementation of ICU practice changes. Construct ratings, ranging in discrete integers from −2 to +2, were determined by the *strength* (reflected by the rating magnitude) and *valence* (reflected by the rating positive/negative sign) of influence each construct had on implementation based on qualitative interview data. Constructs that had mixed comments within a single transcript, that is, comments were equally positive and negative, were assigned a mixed (“0”) rating. Using the rating guidelines outlined in Table 1, the evaluation team achieved high consensus and proceeded to rate the single-coded transcripts individually.

**Table 1.** Criteria used to assign ratings to Consolidated Framework for Implementation Research constructs

Rating	Criteria
−2	The construct has an apparent negative influence on implementing new critical care practices, supported by high agreement between interviewees, strong language, and use of concrete examples.
−1	The construct has a negative influence on implementing new critical care practices. Interviewees make general statements about the construct manifesting in a negative way but without consistent and concrete examples: Construct is mentioned generally but without explicit descriptions of how it manifests Construct has mixed effects, but tipped to a weak negative because the aggregate of the comments indicates an overall negative effect Due to limited data, a general negative effect is inferred based on sufficient information and/or absence of the construct
0	The construct has a mixed influence on implementing new critical care practices, with contradictory comments that are equally positive and negative.
+1	The construct has a positive influence on implementing new critical care practices. Interviewees make general statements about the construct manifesting in a positive way but without consistent and concrete examples: Construct is mentioned generally but without explicit descriptions of how it manifests Construct has mixed effects, but tipped to a weak positive because the aggregate of the comments indicates an overall positive effect Due to limited data, a general positive effect is inferred based on sufficient information and/or presence of the construct
+2	The construct has an apparent positive influence on implementing new critical care practices, supported by high agreement between interviewees, strong language, and use of concrete examples.
N/A	The construct is completely absent from the qualitative interview data.

The summary themes and construct ratings were aggregated by site to develop a case memo for each facility, organized by CFIR construct. Each relevant construct had the following: (1) A summary statement to describe how the construct was generally discussed by interviewees within a site; (2) An aggregate construct rating; (3) Rationale for the rating; and (4) Supporting quotes that exemplified the summary statement and construct rating. The qualitative lead (SS) developed the four memo reports for interview transcripts that underwent double coding. The analysts met to review these case memos and deliberate, discuss, and modify the memos as appropriate. Each analyst independently developed memo reports for the remaining facilities for which single-coding analysis was conducted. The qualitative lead reviewed each memo report and discussed with the analysts to ensure consistency in the data being summarized and recorded across interviews.

**(3) Qualitative comparison of construct ratings between hospital performance groups:** We used framework analysis methods to compare qualitative interview data and the corresponding quantified CFIR construct ratings across sites based on their mortality rates [17]. The analysts were unblinded to facility mortality rate (low and high), and a matrix was created, with facilities sorted in columns by low versus high mortality and constructs identified during the blinded phase listed in rows (see Supplemental Table). Each cell contained information extracted from case memos, including summaries of qualitative data and quantified construct ratings based on all interviews. By grouping sites by low and high mortality, we identified patterns in summaries and ratings of the CFIR constructs that distinguished between sites with low- versus high-mortality rates.

We used predetermined criteria (see Table 2) based on previously described methods [18,19,20,21] to guide the rating analysis protocol and characterize each of the 14 CFIR constructs assessed as (1) a facilitator or barrier (or neither) to the implementation of new critical care practices within low- and high-mortality hospital groups; and (2) a distinguishing (or not distinguishing) construct between low- and versus high-mortality hospital groups. Briefly, we first qualitatively assessed the pattern of positive and negative construct ratings *within* hospital performance groups (low and high mortality) to determine whether the construct facilitated and/or hindered implementation of new critical care practices. In both the low- and high-mortality hospital groups, each construct was categorized as either a *facilitator* (if the median construct rating within the hospital group was *positive*), *barrier* (if the median construct rating within the hospital group was *negative*), or *neither* (if ratings were *mixed* and/or *absent* in qualitative data). Then, we calculated the percentage of facilities within each hospital performance group that were assigned a positive construct rating (either +2, +1, or +.5 for sites with “mixed” ratings) to qualitatively assess differences in construct ratings *between* hospital performance groups (low versus high mortality). As shown in Table 3, constructs were identified as “distinguishing” if the relative difference in positive ratings between hospital performance groups was ≥20% (e.g., 9/9 or 100% of hospitals had positive ratings in low-mortality group and 2/8 or 25% of hospitals had positive ratings in high-mortality group).

**Table 2.** Criteria used to assign implementation influence and distinguishing pattern to Consolidated Framework for Implementation Research constructs

Assignment	Criteria
Implementation influence: the pattern of valence (positive and negative ratings) for each construct *within* hospital performance groups.	
Facilitator	The median construct rating within the hospital group is *positive*, either +1 or +2. If the median rating is +2, characterize as “strong facilitator.”
Barrier	The median construct rating within the hospital group is *negative*, either −1 or −2. If the median rating is −2, characterize as “strong barrier.”
Mixed	The median construct rating within the hospital group is *mixed* (0), and the majority of facilities are assigned mixed ratings.
Absent	The median construct rating within the hospital group is *N/A* (absent from the qualitative interview data), and the majority of facilities are assigned N/A ratings.
Distinguishing pattern: the pattern of construct ratings *between* hospital performance groups.	
Distinguishing	The difference in positive ratings between hospital performance groups is ≥20% (e.g., 9/9 or 100% positive ratings in low-mortality group and 2/8 or 25% positive ratings in high-mortality group).
Not distinguishing	The difference in positive ratings between hospital performance groups is <20% (e.g., 9/9 or 100% positive ratings in low-mortality group and 7/8 or 87.5% positive ratings in high-mortality group).

**Table 3.** Consolidated Framework for Implementation Research construct ratings and implementation influence by hospital performance group

	Low-mortality hospital group		High-mortality hospital group		Distinguishing constructs
	Median rating^a^	Positive rating (%)^b^	Median rating	Positive rating (%)	
I. Intervention characteristics					
Innovation source	+1	89	+1	100	Not distinguishing
Complexity	N/A	N/A	N/A	N/A	Not distinguishing
II. Outer setting					
**Needs of patients**	**+1**	**78**	**+2**	**100**	**Distinguishing**
Cosmopolitanism	+2	100	+2	100	Not distinguishing
III. Inner setting					
**Implementation climate**	−**1**	**11**	**+1**	**56**	Distinguishing*
Tension for change	+1	89	+1	83	Not distinguishing
**Organizational incentives and rewards**	**N/A**	**N/A**	**+1**	**71**	**Distinguishing**
**Leadership engagement**	**+2**	**100**	**+1**	**69**	Distinguishing*
Available resources	+1	83	+1	69	Not distinguishing
Access to knowledge and information	0	50	0	41	Not distinguishing
Networks and communications	+1	89	+1	75	Not distinguishing
IV. Characteristics of individuals					
Knowledge and beliefs about changes	0	49	0	33	Not distinguishing
V. Process					
**Engaging implementation leaders**	**+1**	**100**	**+1**	**75**	**Distinguishing**
**Engaging staff**	**+2**	**100**	**+1**	**75**	Distinguishing*

^a^The median rating within each hospital performance group determined construct implementation influence: **+**1 = facilitator; +2 = strong facilitator; −1 = barrier; −2 = strong barrier; 0 = mixed; N/A = absent.

^b^The percent of positive ratings within each hospital performance group determined construct distinguishing pattern: ≥20% difference in positive ratings between hospital performance groups = distinguishing; < 20% difference in positive ratings between hospital performance groups = not distinguishing.

*Construct has statistically significant differences in ratings between hospital performance groups.

**(4) Quantitative comparison of construct ratings between hospital performance groups:** After identifying qualitative correlations between construct ratings and mortality outcomes, we conducted a Wilcoxon signed rank test to quantitatively identify significant differences in median construct ratings between hospitals with low versus high COVID-19 mortality. All statistical analyses were conducted with R version 4.1.2 (Vienna, Austria) with alpha level = 0.05.

## Results

Facility and interview participant characteristics are previously described [2]. Briefly, 17 facilities across the USA – nine from the lowest adjusted mortality quartile and eight from the highest – agreed to participate among the 20 invited hospitals and varied in size, patient population, management, and funding structure. Across these 17 sites, 31 ICU clinical leaders (15 MDs and 16 RNs) participated in qualitative interviews. Thematic data saturation was achieved. Interview participants varied in age, gender, race, and professional training [2].

### Summary of Findings

Of the 14 CFIR constructs assessed (see Table 3), three constructs (implementation climate; leadership engagement; and engaging staff) had both *qualitative* and statistically significant *quantitative* correlations with mortality outcomes. These three constructs had differences in construct ratings between hospital performance group that were both ≥20% (i.e., “distinguishing” constructs) and statistically significant (*p* < 0.05) according to a Wilcoxon signed rank test. An additional three constructs (needs of patients; organizational incentives and rewards; and engaging implementation leaders) were qualitatively different across mortality outcome groups as “distinguishing” constructs (i.e., differences in qualitatively derived construct ratings between hospital performance groups were ≥20%), but these differences were not statistically significant.

Seven constructs (innovation source; cosmopolitanism; tension for change; available resources; access to knowledge and information; networks and communications; and knowledge and beliefs about changes) did not distinguish between low- versus high-mortality hospitals based on qualitative comparison of construct ratings between low- and high-mortality hospitals (i.e., differences in qualitatively derived construct ratings between hospital performance groups were <20%). In other words, these seven non-distinguishing constructs manifested similarly, as either implementation facilitators, barriers, or mixed influence, in both low- and high-mortality hospitals. The last construct (complexity) had insufficient data to assess qualitative and quantitative differences between hospital groups.

The 13 relevant constructs, their definitions adapted from the CFIR [9], their median and relative positive ratings, and distinguishing patterns are summarized in Table 3 by hospital performance group. The following section describes the qualitative and quantitative associations between mortality outcome (low versus high) and each of the six distinguishing CFIR constructs. The seven non-distinguishing CFIR constructs are outlined in Table 3, but not discussed below.

### CFIR Constructs Associated (Qualitatively and Quantitatively) with Mortality Outcomes

#### Implementation climate

Implementation climate was a distinguishing construct based on qualitative analysis, with a 45% difference in positive ratings between hospital performance groups (11% of low-mortality hospitals versus 56% of high-mortality hospitals). This finding was corroborated by quantitative analysis, which detected a statistically significant difference in median construct ratings (*p* < 0.05) between hospital groups (−1 in the low-mortality group versus +1 in the high-mortality group). Compared to low-mortality sites, high-mortality sites exhibited greater readiness for change, which manifested in more positive construct ratings for "implementation climate." However, within high-mortality sites, this enhanced readiness for change was largely a result of a trial-and-error approach to care with a low evidence threshold for adoption of new critical care practices. While low evidence thresholds promoted an implementation climate in which changes were readily adopted in high-mortality hospitals, they also caused frequent and inconsistent changes to practice, confusion, and disagreement among ICU staff, ultimately associated with worse patient outcomes:

*I felt a lot of it was just trial and error. What was working for one person, maybe not another…"This treatment worked well on this person, can't we try it on this person?" And "Well, they probably won't respond well” …And then sometimes the confusion, if you have a nurse who was there on the Monday and now, they're not back until Friday, and Friday comes and they're like, "Oh, well, we did this on Monday." And you're like, "Okay, well, we're not doing it anymore."* [Nursing director, Facility 1201, high-mortality hospital]

In contrast, low-mortality hospitals reported greater emphasis on identifying and implementing evidence-based practices, even if doing so slowed the adoption of new practices:

*There were multiple discussions with ICU leadership at the outset. And our consensus around the evidence was good. The evidenced-based critical care was what was going to get these patients through the storm, so to speak. So, adhering as best as possible to the protocols, the evidence-based practices, and the standards of care.* [Medical director, Facility 402, low-mortality hospital]

#### Leadership engagement

This construct was distinguished based on qualitative analysis, with a 31% difference in positive ratings between hospital performance groups (100% of low-mortality hospitals versus 69% of high-mortality hospitals). According to quantitative analysis, there was also a statistically significant difference in median construct ratings (*p* < 0.05) between hospital groups (+2 in the low-mortality group versus +1 in the high-mortality group). Low-mortality sites reported having robust high-level hospital leadership presence and direct support on the floor, as well as more engagement from mid-level leaders and providers in the decision-making processes:

*The most helpful [resources to help with making changes] I think was access to the administrative leadership, and being involved in these meetings that I normally am not involved in. I think broadly was the most helpful, because it allowed bi-directional communication, first-hand knowledge.* [Nursing Director, Facility 201, low-mortality hospital]

Contrarily, high-mortality sites cited relatively lower levels of engagement from senior hospital leadership and administration, causing frontline staff to feel unsupported:

*It would’ve meant so much for us to have the leadership walk through our unit. You won't even come in here, but you’re telling me it’s safe for me to do this for hours a day when you won't even walk through the doors of the unit, and you’re telling me it’s okay, but you won't do it yourself. That doesn't fly with me.* [Nursing director, Facility 1301, high-mortality hospital]

#### Engaging staff

Staff engagement by leadership was distinguished based on qualitative analysis, with a 25% difference in positive ratings between hospital performance groups (100% of low-mortality hospitals versus 75% of high-mortality hospitals). According to quantitative analysis, there was also a statistically significant difference in median construct ratings (*p* < 0.05) between hospital groups (+2 in the low-mortality group versus +1 in the high-mortality group). The stronger positive rating of "engaging staff" in the low-mortality group aligns with the qualitative theme emergent in the low-mortality group that implementation of practice changes was facilitated by leaders’ efforts to effectively engage staff through involving them in decisions by soliciting their input on care practices and providing supportive resources to meet their material and personal needs:

*A lot of the decisions I made I didn't make independently either. I consulted with (physician lead) or even with the staff nurses. I would say, “You do the work, tell me what’s going to work. Tell me what you think” …I made it a point to call staff that we sent home. Every day I was calling someone.* [Nursing director, Facility 601, low-mortality hospital]

Conversely, high-mortality hospitals demonstrated lack of leadership attunement to frontline staff needs and voices. This made staff feel disengaged, isolated, and underappreciated, which created a barrier to effectively engaging staff:

*There could have been more intense support of peoples' psychological health and wellbeing. And more thought put into how people are going to manage their issues like childcare, their issues like transport, their issues around how families perceive people who are in close contact with patients and working with them, and things of that nature.* [ICU MD, Facility 1302, high-mortality hospital]

### CFIR Constructs Qualitatively Associated with Mortality Outcome, but Without Statistically Significantly Differences

#### Needs of patients

While both low- and high-mortality sites perceived the commitment to addressing patient and family needs as a facilitator for implementing new care practices, 100% of high-mortality hospitals had a positive construct rating compared to 78% of low-mortality hospitals. The strongly positive median rating of +2 in the high-mortality group, compared to +1 in the low-mortality group, reflects the qualitative theme that high-mortality sites prioritized the "needs of patients" so intensely that they were more likely than low-mortality sites to loosen the visitation policies in order to meet patient and family social and emotional needs:

*It was hard to implement [the visitation policies] because we had patients that were here, several of which did not survive and it’s not customary for us to not allow families to be with their loved ones during that time…So we made exceptions and we accommodated patients so that the patients that needed to have family members were able to be with them.* [Medical director, Facility 902, high-mortality hospital]

Making *ad hoc* policy exceptions to prioritize patient and family needs in high-mortality sites not only challenged implementation of consistent visitation protocols, but may have influenced the safety of staff, patients, and families. Contrarily, low-mortality hospitals prioritized safety by maintaining strict visitation policies, but still met "needs of patients" through improving telecommunication with families:

*We were making sure that daily communication was going out to the family in regards to the overall criticality of the patient…we were also incorporating video communication for those patients that were able to communicate…even patients who were not able to communicate, for example, those who were on respiratory life support, we were utilizing iPads and video to communicate with the family members to show them as best as we could what condition they were in.* [MD, Facility 802, low-mortality hospital]

#### Organizational incentives and rewards

Financial incentives were largely absent in qualitative data from low-mortality hospitals, resulting in a “N/A” median construct rating. In contrast, 71% of high-mortality hospitals scored positive rating and a median rating of +1, reflecting the reported use of financial incentives to accommodate the increased staff workload and retain and attract staff:

*Eventually, [the hospital] came around and they started giving more money to try to keep people here and it worked.* [Nursing director, Facility 1501, high-mortality hospital]

Although extrinsic financial incentives were referenced less frequently in low-mortality hospitals compared to high-mortality ones, low-mortality hospitals cited using non-monetary rewards instead, that is, accolades, shout-outs, and increased stature:

*Several groups and people have received accolades…our nurse manager for a medical ICU was the leader of the month…we've got 17,000 employees. So, it’s a pretty big deal to be a leader of the month. And another one of our nurse managers in the humanities oncology and surgical ICU…was also leader of the month*. [Critical care executive director, Facility 1602, low-mortality hospital]

#### Engaging implementation leaders

Though "engaging implementation leaders" was perceived to facilitate implementation of new critical care practices in both low- and high-mortality hospitals – reflected by the +1 median construct ratings in both groups – 100% of low-mortality hospitals scored a positive construct rating compared to 75% of high-mortality hospitals. Reflected by the widespread positive influence that "engaging implementation leaders" had in low- mortality hospitals, there was a greater emphasis on establishing formal, collaborative, and organized groups (e.g., incident command structures, task forces, special interest groups) in low-mortality group compared to the high-mortality group. Through developing these multidisciplinary communication and network structures, low-mortality hospitals engaged a wide range of implementation leaders during the decision-making and implementation processes and cultivated trusting relationships between stakeholders, thereby facilitating implementation efforts:

*There were people that were key stakeholders across strategic groups across the organization who would be leading teams…all the decisions that we make or the changes that we make to our ICU or anything that we implement actually goes through the team…everyone is involved, everyone has a voice…the trust has grown to a point where these things are really easy to do. Literally, it just took 24 hours to implement our prone positioning protocol which in a pandemic is just testament to how well our processes were.* [Nursing director, Facility 302, low-mortality hospital]

Contrarily, an emerging theme in the high-mortality group was that siloed and authoritarian decision-making from hospital administrators disrupted relationships between hospital leadership and staff and hindered efficient implementation of practice changes:

*Some frustration, of course, that’s expected when you are not involved with the decisions or know why they were made…we as critical care physicians were never involved much to be honest.* [ICU MD, Facility 1502, high-mortality hospital]

## Discussion

This study revealed salient facilitators and barriers to adopting changes in critical care practices during the early COVID-19 pandemic. Beyond influencing the uptake of practice changes, these implementation determinants were associated with patient mortality outcomes. The differences in key contextual elements identified between low- and high-mortality hospitals can inform future implementation strategies.

One of the most compelling differences between low- and high-mortality hospital groups relates to the construct of "leadership engagement." We learned that, compared to high-mortality sites, low-mortality sites had higher levels of hospital leadership accessibility, physical presence, and engagement with frontline ICU staff. Qualitative interview data and resulting strong positive construct ratings suggest that the daily presence and support of mid- and high-level leaders facilitated implementation of new care practices through reducing the overwhelming burdens felt by frontline staff and cultivating a culture of teamwork. Although previous studies have identified leadership engagement as a facilitator of the implementation process [22,23,24], the importance of leadership engagement is further underscored by the association with patient outcomes. Conversely, clinical stakeholders from high-mortality hospitals cited lack of engagement from senior hospital leadership as an implementation barrier and source of frustration among frontline staff.

Another distinguishing construct between low- and high-mortality hospital groups was "engaging implementation leaders," a concept related to "leadership engagement." Low-mortality hospitals tended to engage a broader range of stakeholders, including mid-level leaders and providers in the decision-making and implementation processes. Multi-level and multidisciplinary collaboration has been identified to cultivate inter-professional teamwork, promoting implementation effectiveness by increasing the capacity to problem-solve during uptake of practices [25,26,27]- and encouraging readiness for change among staff [28,29]. In contrast, high-mortality sites reported unilateral and authoritarian decision-making by hospital leaders and administrators removed from frontline care and without collaboration across leadership levels. Major change management strategies utilized by low-mortality sites to engage a wide range of implementation leaders and decision-makers pertained to the "networks and communications" construct. Specifically, low-mortality hospitals fostered multidisciplinary and collaborative networks by establishing formal and organized groups (e.g., incident command structures, task forces, special interest groups), which is an evidence-based practice [30,31] that was largely absent in hospitals in the high-mortality group.

Similar to the substantial distinguishing patterns of the "leadership engagement" and "engaging implementation leaders" constructs, there were both qualitative and statistically significant quantitative differences between low- and high-mortality hospital groups in relation to "engaging staff." Compared to high-mortality sites, low-mortality sites more frequently reported intentional efforts to effectively engage frontline staff throughout the implementation process. A few engagement strategies emphasized by low-mortality sites included soliciting staff input about decisions and practice changes in formal and informal settings and responding to staff concerns and needs through the provision of ample emotional and physical support services. These findings are supported by previous studies, which cite bilateral communication and feedback structures between leadership and staff as effective strategies associated with higher implementation success [32,33,34,35], especially through promoting employee’s receptiveness to change by making them feel valued and essential [36]. Contrarily, both staff feedback and support were lacking across high-mortality sites. Weaker strategies to engage staff in the high-mortality group might explain why these hospitals relied more on "organizational incentives and rewards" to retain dissatisfied staff than low-mortality sites.

Interestingly, the strongest distinguishing construct between high- and low-mortality sites was "implementation climate." Compared to low-mortality sites, high-mortality hospitals reported greater readiness for change, contributing to more positive ratings for this construct. Although this finding was less intuitive than the other results, the qualitative data help reconcile this paradox. Interviews with clinical stakeholders from high-mortality sites revealed that an implementation culture characterized by increased receptiveness to change was largely a consequence of using an indiscriminate trial-and-error approach to adopt new practices. This trial-and-error mindset, which was absent in low-mortality sites, likely resulted from a combination of contextual elements common in high-mortality sites that hindered rigorous implementation of evidence-based practices. For example, organizational barriers include utilization of low evidence thresholds when adopting changes and absence of strong hospital leaders to provide clear guidance on evidence-based practices when available, requiring staff to make *ad hoc* decisions on patient care. Though we cannot infer causation with this study design, these results suggest the possibility that a trial-and-error approach to care without a framework for selecting evidence-based practices and evaluating results may have contributed to worse patient outcomes in the high-mortality hospital group. This theme provides powerful insight on the importance of selective implementation and evaluation of evidence-based approaches, especially in light of recent findings demonstrating that using an intervention readily adopted in many hospitals based on little evidence – use of high-dose vitamin C in sepsis – caused worse outcomes [37].

Another potentially counterintuitive finding was that the "needs of patients" was a distinguishing construct, having stronger positive ratings in the high-mortality versus low-mortality hospital group. Unlike low-mortality sites, high-mortality sites prioritized the "needs of patients," often above the safety of visitors and staff, resulting in the loosening of visitation policies to meet the social and emotional needs of patients and their families. In contrast, low-mortality hospitals prioritized patient, family, and staff safety by maintaining strict visitation policies, but were still committed to meeting the "needs of patients" through implementing robust telecommunication mechanisms between staff and families. Is it also possible that the greater volume of dying patients at high-mortality sites may have prompted the relaxation of policies to allow more families to be at the bedside of dying patients.

## Strengths and Limitations

A strength of this study was the systematic application of CFIR construct ratings that allowed double-blinded collection and quantification of qualitative data. Not only did this rigorous approach enable cross-site quantitative comparisons, but it also allowed us to assess the relative strength and quantitative significance of implementation factors that qualitatively appeared to distinguish between low- and high-mortality hospital groups. In addition to the benefits of quantitative analysis, qualitative data were invaluable in revealing more nuanced differences between hospital groups that the quantified data alone did not capture. Furthermore, the use of CFIR enabled systematic evaluation of facilitators and barriers to implementing changes in healthcare practices across diverse contexts and presentation of findings using common terminology enables comparison across implementation studies.

Our study has several limitations. First, we used the CFIR to guide data analysis but not data collection. Using the CFIR to inform development of the interview guide would have allowed us to intentionally ask participants questions aligned with CFIR constructs, thereby strengthening the theoretical foundation and continuity across data collection, analysis, and interpretation. However, by retroactively mapping each interview question to applicable CFIR constructs [2], we maintained conceptual alignment across data collection and analysis. Additionally, the CFIR lacks the power to investigate interactions between constructs, thereby limiting our ability to assess clustering and confounding effects on the observed associations between individual constructs and hospital mortality rate. Second, due to small sample size, we did not adjust p-values for multiple hypothesis testing. However, given that three constructs had statistically significant (*p* < .05) differences in median ratings between low- and high-mortality hospital groups, it is unlikely that our findings represent false positive results.

The greatest limitation is that findings are based on interviews with a small sample of 31 participants who were chosen for interviews based on their roles in ICU leadership and participated based on willingness to engage in a qualitative interview. Therefore, participants may not be representative of their local facility or other critical care settings. The perspectives of other important stakeholders, including frontline staff and patients, are not captured in these findings. Additionally, the small sample size introduces possible confounding effects of the interview participants themselves on the gradation in hospital performance. However, as with any qualitative data, while our findings may not be generalizable, they provide important information for other critical care sites needing to quickly respond to urgent public health emergencies. The significant impact of this study is that across stakeholders, themes were consistent *within* high and low performers and different *across* high versus low performers. The fact that we obtained thematic saturation within high- versus low-performing hospital groups (i.e., themes clustered and differentiated across hospital performance categories) is notable and suggests that identified institutional commonalities may have influenced mortality rates. While this study cannot prove causation, it should lead to further studies that test whether the implementation practices and factors associated with ICU performance are modifiable and causally related to outcomes.

## Conclusion

This study provides valuable information on the association between organizational implementation constructs and COVID-19 mortality rates. Distinguishing features of low-mortality hospitals such as collaborative and engaged leadership styles can be the focus of implementation efforts across critical care settings. Likewise, constructs differentially represented within high-mortality hospitals such as a trial-and-error approach with low evidence thresholds for implementation indicate areas that can be targeted for mitigation interventions. Future research should evaluate how the constructs differentially associated with patient outcomes can be best harnessed to guide tangible and practical improvements in leadership, communication, and change management structures that contribute to positive clinical outcomes.

## Data Availability

The datasets used and/or analyzed during the current study are not publicly available due to privacy and confidentiality of our research participants, but are available from the corresponding author on reasonable request.

## Supplementary material

For supplementary material accompanying this paper visit https://doi.org/10.1017/cts.2023.22. click here to view supplementary material
