# Supplementary material for: A multisite exploration of the association between critical care implementation factors and clinical outcomes during the COVID-19 pandemic
Source: J Clin Transl Sci. 2023 Feb 17;7(1):e72. doi: 10.1017/cts.2023.22 (PMC10052400; doi:10.1017/cts.2023.22)
Supplement: Supplementary file 1 [file ctssup.zip › S2059866123000225sup001.docx]

**Supplemental materials:**

**Table 1** An example of the matrix of integrated qualitative and quantitative data comparing ratings for CFIR constructs in the inner settings domain across facilities, sorted by low and high mortality rates

| **FACILITY** | **100** | **200** | **300** | **400** | **600** | **700** | **800** | **1600** | **1800** | **900** | **1000** | **1100** | **1200** | **1300** | **1400** | **1500** | **1700** |
| --- | --- | --- | --- | --- | --- | --- | --- | --- | --- | --- | --- | --- | --- | --- | --- | --- | --- |
| **MORTALITY RATE** | **Low** | **Low** | **Low** | **Low** | **Low** | **Low** | **Low** | **Low** | **Low** | **High** | **High** | **High** | **High** | **High** | **High** | **High** | **High** |
| Implementation climate | –1 | –2 | –1 | X | –1 | –2 | –1 | –1 | X | –1 | +1 | +1 | –1 | +1 | X | –1 | +2 |
| Tension for change | +1 | +2 | –1 | +1 | +2 | +1 | +2 | +1 | +1 | +2 | +1 | N/A | –1 | +2 | N/A | +2 | +2 |
| Organizational incentives and rewards | N/A | N/A | N/A | N/A | N/A | X | N/A | +1 | N/A | +1 | +1 | +1 | +1 | –1 | N/A | +1 | –1 |
| Leadership engagement | +2 | +2 | +2 | +1 | +2 | +1 | +1 | +1 | +2 | +1 | +1 | +1 | +1 | –2 | +1 | X | X |
| Available resources | +1 | +2 | +1 | +1 | –1 | +1 | +1 | +1 | X | +1 | +1 | +2 | –1 | –1 | +2 | X | +1 |
| Access to knowledge and information | +1 | X | X | –1 | –1 | –1 | –1 | +1 | +1 | –1 | X | X | –1 | +2 | +2 | –2 | +1 |
| Networks and communications | +1 | +2 | +2 | –1 | +2 | +1 | +1 | +1 | +2 | –1 | +1 | +2 | –1 | +2 | +1 | +1 | +2 |
